# Plasma Levels of Tumor Necrosis Factor-Alpha and Interleukin-6 in Obsessive Compulsive Disorder

**DOI:** 10.1155/2007/65704

**Published:** 2007-02-27

**Authors:** N. Konuk, I. O. Tekın, U. Ozturk, L. Atik, N. Atasoy, S. Bektas, A. Erdogan

**Affiliations:** ^1^Department of Psychiatry, Faculty of Medicine, Zonguldak Karaelmas University, 67600 Zonguldak, Turkey; ^2^Department of Immunology, Faculty of Medicine, Zonguldak Karaelmas University, 67600 Zonguldak, Turkey; ^3^Department of Pathology, Faculty of Medicine, Zonguldak Karaelmas University, 67600 Zonguldak, Turkey

## Abstract

*Aim*. Recent research implicated place of an immune mechanism in the pathophysiology of obsessive-compulsive disorder (OCD). Despite increasing evidence involvement of cytokine release in OCD, results of the studies are inconsistent. The aim of this study was to evaluate the plasma levels of the cytokines; tumor necrosis factor-alpha (TNF-*α*) and interleukin-6 (IL-6) in OCD patients. *Methods*. Plasma concentrations of TNF-*α* and IL-6 were measured in 31 drug-free outpatients with OCD, and 31-year age and sex-matched healthy controls. TNF-*α* and IL-6 concentrations in blood were determined by enzyme-linked immunosorbent assay (ELISA). *Results*. Both TNF-*α* and IL-6 levels showed statistically significant increases in OCD patients compared to controls (*P* < .000, *P* < .001, resp.). In addition, the age of onset was negatively correlated with TNF-*α* level (*r* = −.402,* P* = .025) and duration of illness was weakly correlated with IL-6 levels (*r* : .357; *P* : .048) in patients group. *Conclusion*. OCD patients showed increases in TNF-*α* and IL-6 levels compared to the healthy controls. This study provides evidence for alterations in the proinflamatory cytokines which suggest the involvement of the immune system in the pathophysiology of OCD.

## 1. INTRODUCTION

Previous studies implicated the role of the immune system in the pathogenesis of a variety of neuropsychiatric disorders, including depression [1], dementia [2], and schizophrenia [3]. Various immune parameters have also been investigated in some anxiety disorders such as posttraumatic stress disorder [4], panic disorder [5], social phobia [6], and OCD. Recent reports have indicated the presence of immune system alterations in OCD patients. Despite the strong recent interest in immunologic abnormalities in OCD, few studies have examined cytokines in this disorder [7–9].

OCD is characterized by intrusive, unwanted, and recurrent thoughts (obsessions) and/or repetitive ritualistic behaviors (compulsions). It has been suggested that proinflammatory cytokines are involved in the etiopathogenesis of OCD. These results suggest the existence of a possible immune dysfunction in OCD. While some authors reported decreases of TNF-*α*, IL-6, and natural killer (NK) activities [8–11], others found increase of NK cells in OCD patients [12].

An association of OCD with infectious disease and alterations in immune function was first considered when people who had recovered from von Economo's encephalitis began to present neuropsychiatric symptomps. In addition recent research revealed an association between OCD and streptococcal infections. It has been hypothesized that subgroup of children with OCD develops the illness following infection with Group A *β*-hemolytic streptococcus [13].

The most extensively investigated cytokines in neuropsychiatric disorders are TNF-*α* and IL-6 due to their effects on central nervous system (CNS). TNF-*α* is produced by macrophages and circulating monocytes, and plays an important role in a variety of infectious, inflammatory, and autoimmune conditions [14]. TNF-*α* also affects central processes directly or indirectly through stimulation of vagal afferents [15–17]. Thus, this cytokine has been emerging as an important role of the CNS function [18]. IL-6 acts on a variety of cells, regulating immune response, acute phase reaction, and is implicated in the pathogenesis of autoimmune and inflammatory disease [19]. IL-6 is synthesized and colocalized with its own receptors in the brain, and it is expressed in small quantities in the CNS even in the absence of inflammation [20, 21].

Although several studies have suggested the existence of an association between OCD and cytokine levels alteration, the findings are not consistent. Therefore, a thorough investigation of the immune system function in OCD is still warranted. In this study we have measured plasma concentrations of IL-6 and TNF-*α* in OCD patients, with the aim to see whether or not the release of these two proinflammatory cytokines is altered and if they correlate with the type of psychopathology, severity, age of onset, and duration of illness.

## 2. MATERIAL AND METHODS

### 2.1. Subjects

The study was conducted between October 2004 and July 2006. All patients were recruited from the psychiatry outpatients unit of Zonguldak Karaelmas Medical Faculty Hospital. The Local Ethic Commitee approved the study protocol. All subjects were asked to participate and they provided written informed consent. At the initial assessment, the study group was evaluated by psychiatrists (authors) using the Structured Clinical Interview for DSM-IV, Clinical Version (SCID-I/CV) [22, 23].

Subjects were excluded if they had evidence of traumatic injury, clinically unstable medical illness such as hepatic or renal impairment; a history of seizure, head trauma, or stroke, active infection, allergy, rheumatoid disease, cancer, and any other primary disease interfering with immune functions. The patients who were using psychotropic agents (antidepressants, anxiolytics, antipsychotics) and/or analgesics (including nonsteroidal anti-inflammatory drugs) within the last 3 months were excluded from the study. Patients who had history of alcohol or substance abuse and heavy cigarette smoking were also excluded. Patients smoking cigarettes more than 20 per day were considered heavy smokers.

Fifty-three consecutive patients with OCD were included in the study. Ten patients were excluded since they were not drug-free at baseline, six patients excluded since they had the medical illness mentioned above, and five refused to participate. One patient excluded because of extreme value for IL-6. The final sample encompassed 31 patients (17 females and 14 males, mean age 33.4 ± 10.9 years, range 21–64 years). Six of the patients were drug-free and 25 of patients were drug-naive at the study entrance. Of the patients 38.7% had a depressive disorder and 35.4% had comorbid anxiety disorders. The gender and age matched 31 healthy comparison subjects were recruited from the university hospital staff and friends of the staff members (15 females and 16 males, mean age 32.7 ± 8.5 years, range 19–60 years) as controls.

The severity of symptoms was assessed by means of the Yale Brown Obsessive-Compulsive Scale (Y-BOCS), [24]. The levels of depression and anxiety were also assessed using the Hamilton Anxiety Rating Scale [25] and 17-item Hamilton Depression Rating Scales [26], which are rated by physicians. These scales have been demonstrated to be valid and reliable in Turkish population studies [27, 28].

### 2.2. Elisa

Ten ml of heparinized venous blood were collected with the plastic tubes from subjects at 08.00 a.m. Blood samples were santrifuged at 3000 rpm (rotor diameter: 16 cm) and preserved at −80°C. Analyses were performed by the immunologists, who were blind to the condition of the samples. IL-6, TNF-*α* enzyme-linked immunosorbent assay (ELISA) kits were purchased by Biosource International Inc.(Camarillo, Calif, USA) and used according to the recommendations of the manufacturer. The minimum detectable doses of TNF-*α* and IL-6 are 1.1 pg/ml, 2.2 pg/ml, respectively. There is no cross-reactivity with other cytokines. All samples were assayed in duplicate.

### 2.3. Statistics

Results were analyzed at the computer by using the Statistical Package for the Social Sciences for Windows release 11.01, Chicago Illionis (customer no. 114094). Data were expressed as mean ± standard deviation. The Kolmogorov-Smirnov test was used to evaluate the normality of the data for OCD patients (TNF-*α*; *P* = .031, IL-6: *P* = .000). Patients and controls test scores were compared by the Mann Whitney U test as the data not distributed normally. The differences were considered to be significant when the *P* value was less than .05. All tests performed were two-tailed. In addition Spearman correlation tests were performed in order to test intercorrelations between clinical findings such as Y-BOCS total and subscale scores age, age at onset, duration of illness, and cytokine levels in OCD group.

## 3. RESULTS

Demographic and clinical characteristics of the patients are shown in Table 1. There were a total of 62 subjects, including 31 patients (17 females and 14 males) and 31 healthy control subjects (15 females and 16 males). The mean ages (± standard deviation) of the patients and control subjects were 33.4 (±10.9) and 32.7 (±8.5) years, respectively. There were no significant group differences between patients and controls for age and gender (*P* > .05). The mean age at onset of obsessive-compulsive symptoms was 22.8 ± 10.7 years, with a length of illness of 10.6 ± 10.6 years at entry. The Y-BOCS total score was 23.3 ± 8.8 (minimum 8, maximum 40), the obsession subscale total score was 11.9 ± 4.0, and the compulsion subscale total score was 11.4 ± 5.2. The most commonly reported obsessions were aggressive (78.9%), and the most common compulsions were cleaning (68.7%) and checking (46.9%).

Differences in immune system variables between the OCD patients and control subjects are shown in Table 2.

The age of onset was negatively correlated with TNF-*α* level (*r* = −.402, *P* = .025). There was positive weak correlation between duration of illness and IL-6 levels (*r* : .357; *P* : .048). No other correlations were found between clinical features with any of the immune parameters.

When group was reanalyzed in terms of an onset of the disease, patients with an early onset before age 14 had significantly higher TNF-*α* levels than patients with nonearly onset (*P* = .018). Nonparametric Kruskal Wallis tests with adjustment by Bonferroni correction were conducted to evaluate whether the differences were significant between two compared groups (*P* < .0016) (early onset, nonearly onset and control groups). Levels of TNF-*α* (16.8 ± 9.0, 9.9–39.2) and IL-6 (16.9 ± 16.0, 6.5–54.9) were significantly increased in early onset group compared with nonearly onset and healthy controls (Kruskal Wallis, Asymp. Sig. *P* < .000 for both TNF-*α* and IL-6). In contrast there was no significant increase in TNF-*α* and IL-6 between nonearly onset and healthy controls.

## 4. DISCUSSION

In our investigation TNF-*α* and IL-6 plasma levels were significantly higher in OCD patients compared to healthy controls. Our results corroborate the findings of previous studies on immune alteration in OCD [7–13]. However to date in the literature there have been limited studies on alterations of cytokines and results are conflicting. To our medline search we reached only four studies investigating plazma levels of TNF-*α* in OCD patients. The observation of increased TNF-*α* production in present study is not in accordance with these five study results that found the decreased TNF-*α* plasma levels in patients with OCD [9–11, 29]. TNF-*α* is one of the main cytokines in the inflammatory and immune responses. The alterations of serotonergic pathways have long been evidenced in OCD [30].

It has been demonstrated that TNF-*α* may provoke variations of central neurotransmitter activity, and conversely that neurotransmitters may modulate expression. Enhancement of serotonin transporter function by TNF-*α* also has been reported by Mössner et al. [31]. Moreover, possible induction of TNF-*α* expression by serotonin has been shown in rat hippocampal astrocytes [31, 32]. Since existence of multidirectional communication among the immune system and the central nervous system has been shown, the alteration in immune function in OCD would reflect a change in neurotransmission.

Study results of plasma levels of IL-6 have also been conflicting. Although most of the studies have not found an alteration in IL-6 plasma levels in OCD [7, 11, 33] we detected an increase in IL-6 levels. Inconsistencies in cytokine measurements may be due to differences in methodologies. For example sampling of CSF rather than plasma in the study of Carpenter et al. [33] may be the cause of their negative findings. As plasma levels of IL-6 measured only once in present study which cannot reflect the values of IL-6 throughout the day might be explained the differences between results.

Since TNF-*α* and IL-6 are major proinflammatory cytokines and our findings of higher blood levels of these two cytokines support the evidence of immune activation in OCD that ongoing immune activation may be involved in the pathogenesis of OCD, most studies that have reported a normal, or an increase of proinflammatory cytokines rather than a decrease in other psychiatric disorders, namely, major depression and schizophrenia, support this hypothesis [34–36]. Moreover increased prevalence of autoimmune diseases and of antinuclear and anticytoplasmic antibodies increased serum IL-6 concentration, and an association with HLA antigens has been found in schizophrenic patients [37]. These findings are also characteristic of apparent autoimmune diseases such as systemic lupus erythematosus, rheumatoid arthritis. Although the high serum levels of TNF-*α* and IL-6 in the OCD patients of our study could be suggestive of an ongoing autoimmune process, the mechanism underlying these altered cytokine levels remains unknown at present. Since the triggering of an autoimmune process depends on the inappropriate action of certain cytokines affecting various immunologic functions, studies in OCD patients assessing the levels of other cytokines or cellular responses are needed.

Another important observation in the present study was that clinical variables of OCD were related to specific alterations in immune parameters. The increased IL-6 production in our sample may still be a characteristic of a particular subgroup of OCD patients as speculated in the literature [38], or a state factor of severely ill OCD patients, as we found correlation between duration of illness and IL-6 production. We have found, in addition, that patients with a childhood onset of OCD (<14 years) had a higher level of TNF-*α* than patients with nonearly onset. Immunologic alterations appear to be different in children and adult patients probably reflect different pathophysiologic mechanisms, such as autoimmunity. We have not found any other correlation between cytokines and severity of the disease, existence of comorbid depression, anxiety, and family history of OCD.

Inclusion of the untreated OCD patients in this study might explain the discrepant results from other studies. The evidence suggests that drugs such as benzodiazepines [39], antidepressants [40], and antipsychotics [41] may have significant influence on the functioning of immune system. Several studies reported normalization of the changes in a number of NK cells and level of IL-6 in depressed patients following chronic selective serotonin reuptake inhibitors treatment [42]. However, Barber et al. did not observe any change in T-lymphocyte subsets during clomipramine treatment in chronic OCD patients [43]. Olanzapine, an atypical antipsychotic, which shares similarities with clozapine in its chemical structure and neurotransmitter receptor binding profiles, has also been shown to cause cellular immune impairment [41]. Interestingly these drugs also induce obsessive compulsive symptoms in a portion of psychiatric patients during the course of treatment [44].

Higher number of comorbidity in our study sample (especially presence of comorbid depressive disorder) could be taken into account to explain the diversity of the results. In earlier studies, depression alone is shown to result in changes in immune functioning of the patients [45]. The findings of present study are in line with reports indicating an increase in the production or secretion of IL-6 and IL-l in subjects with major depression [42]. However, we were not able to find any correlation between existence of comorbid depression or HAM-D scores of the patients and cytokine levels.

Since the size of present study groups was relatively small, we could not claim that an alteration could be detected in all OCD patients. Also as plasma levels of these cytokines measured only once, we cannot decisively say that values are higher than average levels throughout the day. It is possible, however, that cytokines other than IL-6 and TNF-*α* might be involved in this process which constitutes another limitation of this study, as we were unable to conduct assays for additional cytokines.

It has been postulated that OCD may be a manifestation of poststreptococcal autoimmunity, and it is known that individuals with autoimmune pathology or systematic autoimmune diseases often exhibit increased production of proinflammatory cytokines such as IL-6 and TNF-*α* [46]. Our observations confirm an increased IL-6 and TNF-*α* production, however contradict decreased production of proinflammatory cytokines, support the postulation of an autoimmune mechanism in OCD. Since the complex network of processes responsible for the regulation of cytokines is not proven and they have various biological activities, their significance as regulators of physiology of the brain has not well understood yet, determining the significance of the alterations in immune activity in OCD is needed.

## 5. CONCLUSION

In conclusion, our results show an increased production of the proinflammatory cytokines, IL-6, and TNF-*α* in OCD patients. Based on the recent findings of immune abnormalities in the pathogenesis of OCD [47, 48], the present findings are of considerable significance that highlights the needs of further research in this area. Understanding of alteration of cytokine profile in the OCD may help elucidate the role of inflammation and also lead to the design of new therapeutic modalities for this disease.

## Figures and Tables

**Table 1:** Demographic and clinical characteristics of the subjects.

	Patients	Controls

Age	33.4 ± 10.9	32.7 ± 8.5

Gender (female/male)	17/14	15/16

Age at onset	22.8 ± 10.7	—

Duration of illness	10.6 ± 10.6	—

Y-BOCS-obsession	11.9 ± 4.0	—

Y-BOCS-compulsion	11.4 ± 5.2	—

Y-BOCS-total	23.3 ± 8.8	—

HAM-D score	9.9 ± 6.5	—

HAM-A score	21.0 ± 10.7	—

Comorbid depression	12/31	—

Comorbid anxiety	11/31	—

Family history of OCD	5/31	—

Number of early onset OCD patients	9/31	—

**Table 2:** TNF-*α* and IL-6 levels in the patients with obsessivecompulsive disorder and control group.

	OCD patients	Healthy controls	*P* value

TNF-*α* (pg/ml)	13.7 ± 10.61	7.2 ± 3.37	*P* < .000

IL-6 (pg/ml)	15.2 ± 20.6	7.0 ± 1.39	*P* < .001

## References

[1] Connor TJ, Leonard BE (1998). Depression, stress and immunological activation: the role of cytokines in depressive disorders. *Life Sciences*.

[2] Leonard BE (2001). Changes in the immune system in depression and dementia: causal or co-incidental effects?. *International Journal of Developmental Neuroscience*.

[3] Hinze-Selch D, Pollmächer T (2001). In vitro cytokine secretion in individuals with schizophrenia: results, confounding factors, and implications for further research. *Brain, Behavior, and Immunity*.

[4] Maes M, Lin A-H, Delmeire L (1999). Elevated serum interleukin-6 (IL-6) and IL-6 receptor concentrations in posttraumatic stress disorder following accidental man-made traumatic events. *Biological Psychiatry*.

[5] Brambilla F, Bellodi L, Perna G (1999). Plasma levels of tumor necrosis factor-alpha in patients with panic disorder: effect of alprazolam therapy. *Psychiatry Research*.

[6] Rapaport MH, Stein MB (1994). Serum interleukin-2 and soluble interleukin-2 receptor levels in generalized social phobia. *Anxiety*.

[7] Maes M, Meltzer HY, Bosmans E (1994). Psychoimmune investigation in obsessive-compulsive disorder: assays of plasma transferrin, IL-2 and IL-6 receptor, and IL-1*β* and IL-6 concentrations. *Neuropsychobiology*.

[8] Weizman R, Laor N, Barber Y (1996). Cytokine production in obsessive-compulsive disorder. *Biological Psychiatry*.

[9] Brambilla F, Perna G, Bellodi L (1997). Plasma interleukin-1*β* and tumor necrosis factor concentrations in obsessive-compulsive disorders. *Biological Psychiatry*.

[10] Denys D, Fluitman S, Kavelaars A, Heijnen C, Westenberg H (2004). Decreased TNF-*α* and NK activity in obsessive-compulsive disorder. *Psychoneuroendocrinology*.

[11] Monteleone P, Catapano F, Fabrazzo M, Tortorella A, Maj M (1998). Decreased blood levels of tumor necrosis factor-alpha in patients with obsessive-compulsive disorder. *Neuropsychobiology*.

[12] Ravindran AV, Griffiths J, Merali Z, Anisman H (1999). Circulating lymphocyte subsets in obsessive compulsive disorder, major depression and normal controls. *Journal of Affective Disorders*.

[13] Swedo SE, Leonard HL, Mittleman BB (1997). Identification of children with pediatric autoimmune neuropsychiatric disorders associated with streptococcal infections by a marker associated with rheumatic fever. *American Journal of Psychiatry*.

[14] Vassalli P (1992). The pathophysiology of tumor necrosis factors. *Annual Review of Immunology*.

[15] Hopkins SJ, Rothwell NJ (1995). Cytokines and the nervous system I: expression and recognition. *Trends in Neurosciences*.

[16] Rothwell NJ, Hopkins SJ (1995). Cytokines and the nervous system II: actions and mechanisms of action. *Trends in Neurosciences*.

[17] Fiore M, Alleva E, Probert L, Kollias G, Angelucci F, Aloe L (1998). Exploratory and displacement behavior in transgenic mice expressing high levels of brain TNF-*α*. *Physiology and Behavior*.

[18] Maier SF, Watkins LR (1998). Cytokines for psychologists: implications of bidirectional immune-to-brain communication for understanding behavior, mood, and cognition. *Psychological Review*.

[19] Kishimoto T (1992). Interleukin-6 and its receptor in autoimmunity. *Journal of Autoimmunity*.

[20] Schobitz B, de Kloet ER, Sutanto W, Holsboer F (1993). Cellular localization of interleukin 6 mRNA and interleukin 6 receptor mRNA in rat brain. *European Journal of Neuroscience*.

[21] Schöbitz B, de Kloet ER, Holsboer F (1994). Gene expression and function of interleukin 1, interleukin 6 and tumor necrosis factor in the brain. *Progress in Neurobiology*.

[22] First MB, Spitzer RL, Gibbon M, Williams JBW (1997). *Structured Clinical Interview for DSM-IV Clinical Version (SCID-I/CV)*.

[23] Özkürkçügil A, Aydemir Ö, Yıldız MK, Esen Danacı A, Köroğlü E (1999). DSM IV Eksen I bozuklukları için yapılandırılmış klinik görüşmenin Türkçe'ye uyarlanması ve güvenilirlik çalışması. *İlaç ve Tedavi Dergisi*.

[24] Goodman WK, Price LH, Rasmussen SA (1989). The Yale-Brown obsessive compulsive scale. II. Validity. *Archives of General Psychiatry*.

[25] Hamilton M (1960). A rating scale for depression. *Journal of Neurology, Neurosurgery, and Psychiatry*.

[26] Hamilton M (1959). The assessment of anxiety states by rating. *The British Journal of Medical Psychology*.

[27] Akdemir A, Türkçapar MH, Örsel SD, Demirergi N, Dag I, Özbay MH (2001). Reliability and validity of the Turkish version of the Hamilton Depression Rating Scale. *Comprehensive Psychiatry*.

[28] Yazıcı MK, Demir B, Tanrıverdi N, Karaağaoğlu E, Yolaç P (1998). Hamilton Anksiyete Değerlendirme Ölçeği, değerlendiriciler arası güvenirlik ve geçerlik çalışması. *Türk Psikiyatri Dergisi*.

[29] Mittleman BB, Castellanos FX, Jacobsen LK, Rapoport JL, Swedo SE, Shearer GM (1997). Cerebrospinal fluid cytokines in pediatric neuropsychiatric disease. *Journal of Immunology*.

[30] Zohar J, Kennedy JL, Hollander E, Koran LM (2004). Serotonin-1D hypothesis of obsessive-compulsive disorder: an update. *The Journal of Clinical Psychiatry*.

[31] Mössner R, Heils A, Stöber G, Okladnova O, Daniel S, Lesch K-P (1998). Enhancement of serotonin transporter function by tumor necrosis factor alpha but not by interleukin-6. *Neurochemistry International*.

[32] Brebner K, Hayley S, Zacharko R, Merali Z, Anisman H (2000). Synergistic effects of interleukin-1*β*, interleukin-6, and tumor necrosis factor-*α*: central monoamine, corticosterone, and behavioral variations. *Neuropsychopharmacology*.

[33] Carpenter LL, Heninger GR, McDougle CJ, Tyrka AR, Epperson CN, Price LH (2002). Cerebrospinal fluid interleukin-6 in obsessive-compulsive disorder and trichotillomania. *Psychiatry Research*.

[34] Kronfol Z, Remick DG (2000). Cytokines and the brain: implications for clinical psychiatry. *American Journal of Psychiatry*.

[35] Lanquillon S, Krieg J-C, Bening-Abu-Shach U, Vedder H (2000). Cytokine production and treatment response in major depressive disorder. *Neuropsychopharmacology*.

[36] Theodoropoulou S, Spanakos G, Baxevanis CN (2001). Cytokine serum levels, autologous mixed lymphocyte reaction and surface marker analysis in never medicated and chronically medicated schizophrenic patients. *Schizophrenia Research*.

[37] Ganguli R, Brar JS, Chengappa KNR, Yang ZW, Nimgaonkar VL, Rabin BS (1993). Autoimmunity in schizophrenia: a review of recent findings. *Annals of Medicine*.

[38] Tükel R, Ertekin E, Batmaz S (2005). Influence of age of onset on clinical features in obsessive-compulsive disorder. *Depression and Anxiety*.

[39] Zavala F (1997). Benzodiazepines, anxiety and immunity. *Pharmacology and Therapeutics*.

[40] Maes M (2001). The immunoregulatory effects of antidepressants. *Human Psychopharmacology*.

[41] Bilici M, Tekelioğlu Y, Efendioğlu S, Ovali E, Ülgen M (2003). The influence of olanzapine on immune cells in patients with schizophrenia. *Progress in Neuro-Psychopharmacology and Biological Psychiatry*.

[42] Sluzewska A, Rybakowski JK, Laciak M, Mackiewicz A, Sobieska M, Wiktorowicz K (1995). Interleukin-6 serum levels in depressed patients before and after treatment with fluoxetine. *Annals of the New York Academy of Sciences*.

[43] Barber Y, Toren P, Achiron A (1996). T cell subsets in obsessive-compulsive disorder. *Neuropsychobiology*.

[44] Lykouras L, Zervas IM, Gournellis R, Malliori M, Rabavilas A (2000). Olanzapine and obsessive-compulsive symptoms. *European Neuropsychopharmacology*.

[45] Maes M (1995). Evidence for an immune response in major depression: a review and hypothesis. *Progress in Neuro-Psychopharmacology and Biological Psychiatry*.

[46] Petitto JM, Repetto MJ, Hartemink DA (2001). Brain-immune interactions in neuropsychiatry: highlights of the basic science and relevance to pathogenic factors and epiphenomena. *CNS Spectrums*.

[47] Murphy TK, Petitto JM, Voeller KK, Goodman WK (2001). Obsessive compulsive disorder: is there an association with childhood streptococcal infections and altered immune function?. *Seminars in Clinical Neuropsychiatry*.

[48] Murphy TK, Sajid MW, Goodman WK (2006). Immunology of obsessive-compulsive disorder. *Psychiatric Clinics of North America*.

